# Supplementary material for: Patterns of Intron Gain and Loss in Fungi
Source: PLoS Biol. 2004 Nov 30;2(12):e422. doi: 10.1371/journal.pbio.0020422 (PMC532390; doi:10.1371/journal.pbio.0020422)
Supplement: Table S1 — Also available at http://genes.mit.edu/NielsenEtAl/. (4.3 MB ZIP). [file pbio.0020422.st001.zip › NielsenEtAl/html/326.html]

AN6703.1.NCU07607.1.MG09827.1.FG04204.1


```
 CLUSTAL W (1.82) Multiple Sequence Alignments - Introns Inserted


Sequence 1: MG09827.1	515 aa
Sequence 2: FG04204.1	482 aa
Sequence 3: NCU07607.1	557 aa
Sequence 4: AN6703.1	495 aa
Alignment Length: 559 aa
Number Identitical Residues: 256 aa
Alignment Score (without introns) 12366


MG09827.1 	-----------------MEDTEKPSTEHAGTVRRTDEA--DLPPWHGMSAGRYLATRVSS
NCU07607.1	MASPDPEKAGSPGHGLGGMDHSHQYTENNGMNTIIDENGHEQPPHHGMSIGQYVKTRFTT
FG04204.1 	------------------------MTQDEYNTR---------APHEGMSPGRYVATRLSS
AN6703.1  	-------------------MADDQVKAQAGHIEAPEEH----RPLTFKACFQYLKDRVPT
          	                     .   . .       ..      *    :  :*:  *..:

MG09827.1 	LKPPMESAPNPLKLLLMLDKHCWAFFM~VAFLAW0TWDAFDFFTVSLTVSDLAKDFGRTN
NCU07607.1	LKPAMRPAPNPIRLLRMITGRQWAFFG~VAFFAW0TWDAFDFFTVSLTVEELAKDFGKTN
FG04204.1 	LKPPMHNVPNPIKLLRMLNSQQWAFFF~IAFAAW0TWDAYDFFSVSLTVTDLAETFGKSK
AN6703.1  	LKPPMRKAPNPFKALTLLNRQQWLFFA0VAFLGW~TLDALDFFTVSLTTSELAEEFGKEI
          	***.*. .***:: * ::  : * **  :** .* * ** ***:****. :**: **:  

MG09827.1 	TDITWGITLVLMFRSVGSITFGIASDRYGRKWPFIVNNLLFIVLEL0GTGFCNTYSQFLA
NCU07607.1	TDITWGITLVLMLRSIGAISFGIAADRYGRKWPFVVNNLLFIVLEL0GTAFSQTYSQFLA
FG04204.1 	KDITWGITLVLMFRSVGSITFGIASDRYGRKWPFVINNLLFIVLEL0----------FLA
AN6703.1  	SDISWGITLVLMLRSVGAITFGIASDRWGRKWPFIINNLLFIALEI~GTGFTQTYQQFLA
          	.**:********:**:*:*:****:**:******::******.**: .:.  .: ..***

MG09827.1 	CRALFGIAMGGLYGNAAATALEDLPEEARGLMSGLLQQG0YAFGYLLATAFARALVNTTP
NCU07607.1	CRALFGIAMGGLYGNAAATALEDLPPAARGLMSGLLQQG~YAFGYLLATAFARGLVNTTS
FG04204.1 	CRALFGVAMGGLYGNVAATALEDCPEEARGLISGMLQQG0YAFGYLLCTAFARGLVDTTS
AN6703.1  	VRALFGIAMGGLYGNVAATALEDCPKEARGIVSGLLQQG~YAFGYLLAAAFARALVGTTS
          	 *****:********.******* *  ***::**:**** *******.:****.**.**.

MG09827.1 	YGWRPLFWFGACPPAIFIAFRLLLPETRMYQQQAEMRQAAAASGTS---VSKTFVAEGKV
NCU07607.1	HGWRPLFWFGACPPVLIIALRLLLPETEIYEQHASVRRTARANGHDKGGVSATFLREGKV
FG04204.1 	HGWRPLFWFGACPPALFIIWRLMLPETQAYQERQHLRQEAGADGK-----GKVFIKEGKV
AN6703.1  	HGWRPFYWFAGCLPVLAIVFRLFLPETNAYRQRQELRAEMPGNVTS------AFISEGKV
          	:****::**..* *.: *  **:****. *.::  :*    ..  .      .*: ****

MG09827.1 	ALRRHWMILLYLVLLMAGFNFM~SHGSQDLYPTMLSNQFGFGPNDVTITQIVANLGAILG
NCU07607.1	AIKKHWLILIYLVLLMAGFNFM~SHGSQDLYPTMLRSQYSFSANAVTVTQVVANLGAMSG
FG04204.1 	ALKHHWLLLVYLVLLMAGFNFM~SHGSQDLYPTMLENQLNFSKNKVTVTQVVANLGAMTG
AN6703.1  	ALKRHWLLLIYLVLLMAGFNFM0SHGSQDLYPTLVQRQYGFSRDAVTVTQVVANLGALTG
          	*:::**::*:************ **********::  * .*. : **:**:******: *

MG09827.1 	GTTVGYCSQIFGRRLSII~VMCIVGGALLYPYTFVDTRAVAAAAFFEQFCVQGAWGVIPI
NCU07607.1	GIIVGFASQIFGRRISII~VMCIIGGALLYPYTFISSKRLIAAAFFEQFAVQGAWGVIPI
FG04204.1 	GSVVGFLSQSVGRRISII0VTNFPN---------------HSNGVLMPCSVQGAWGVIPI
AN6703.1  	GTLCGWGSQIFGRRFSII~VISIVGGALLYPYTFISSHKIMAPAFFEQFCVQGAWGVIPI
          	*   *: ** .***:*** *  : ..:   . :  .:    : ..:   .**********

MG09827.1 	HLMELSPGAFRTFVVGTSYQLGNLVSSASSTIESSIGERFPLPANPGSTENRFEYGKVVC
NCU07607.1	HLMELSPGAFRTFVVGTSYQLGNLVSSASSTIESTIGERFPLENG--GGKVGYEYGKVIC
FG04204.1 	HLMELSPGAFRTFVVGTSYQLGNLVSSASSTIEATIGERFPLPDN-AEGESRYDYGKVIC
AN6703.1  	HLMELSPGSIRTFTVGTAYQLGNLVSSASSTIESTIGERFPLPPT-EEGEERYDYGKVIC
          	********::***.***:***************::*******       :  ::****:*

MG09827.1 	IFMGCVYIYTIILTLIGPEHLKRKFDVDHDEDAKEVMAETGV------VVG-----EGTA
NCU07607.1	IFMGCVYVYTILLTFVGPEYLGRSFDVSEDEDVREVAGEEVVRGAVIASHMHGHGHGGDA
FG04204.1 	IFMACVYVYVIVLTFVGPENLRGKFDVAHDADTREVMGTRAM------DHADQYRRSGDE
AN6703.1  	IFMGCVYAYVIIVTFFGPERLGRDFDVENDEDVGEVRAERGV-----------------I
          	***.*** *.*::*:.*** *  .*** .* *. ** .   :                  

MG09827.1 	ASHVGGAKGKGEIV-----------
NCU07607.1	EGSGSGSEGGEGVGGKAQGKEVEVA
FG04204.1 	ENFRKDSTEKPEVAHVRD-------
AN6703.1  	DDMEKGRN-----------------
          	 .   .
```
